# Unveiling the role of circulating monocyte-macrophage polarization state and PD-L1/PD-L2 expression in newly diagnosed pediatric ITP

**DOI:** 10.1007/s44313-026-00163-0

**Published:** 2026-10-01

**Authors:** Asmaa M Zahran, Omnia El-Badawy, Mervat A. M. Youssef, Mena Sameh Yousef Ghattas, Maha H. Mohamed, Salma G. Morsy, Khalid I. Elsayh, Hossam Elashmawy, Zeinab Albadry M. Zahran

**Affiliations:** 1 Clinical Pathology Department, South Egypt Cancer Institute, Assiut University, Assiut, Egypt; 2 Department of Medical Microbiology & Immunology, Faculty of Medicine, Assiut University, Assiut, Egypt; 3 Department of Basic Medical Sciences, Badr University in Assiut (BUA), Assiut, Egypt; 4 Pediatric Department, Hematology Unit, Children’s Hospital, Faculty of Medicine, Assiut University, Assiut, Egypt; 5 School of Biotechnology, Badr University in Assiut (BUA), Assiut, Egypt; 6 Department of Cancer Biology, South Egypt Cancer Institute, Assiut University, Assiut, Egypt; 7 Pediatric Department, Faculty of Medicine, Assiut University, Hematology Unit, Assiut, Egypt; 8 Clinical Pathology Department, Faculty of Medicine, Al Azhar University, Assiut, Egypt; 9 Clinical Pathology Department, Faculty of Medicine, Assiut University, Assiut, Egypt

**Keywords:** Pediatric, ITP, Monocyte-macrophage polarization, SDG3

## Abstract

**Background:**

Immune thrombocytopenia (ITP) is an immune-mediated disorder characterized by decreased platelet count resulting from immune dysregulation and altered cellular immune responses. This study aimed to evaluate monocyte–macrophage polarization patterns and the expression of immune checkpoint molecules CD273 (PD-L2) and CD274 (PD-L1) in patients with ITP and explore their association with hematological parameters and response to corticosteroid therapy.

**Methods:**

This case–control study compared patients with primary ITP and healthy controls. Hematological indices were assessed using complete blood counts, and monocyte–macrophage subsets including M1-like and M2-like populations and their subtypes (M2a, M2b, and M2c), along with CD273 and CD274 expression were analyzed by flow cytometry.

**Results:**

Patients with ITP showed significantly lower hemoglobin levels, platelet counts, and lymphocyte counts, with higher mean platelet volume (MPV) and platelet distribution width (PDW) compared with controls. A marked increase in M1-like monocyte–macrophages with a concomitant reduction in total M2-like cells was observed in patients with ITP. Within M2 subsets, M2a-like cells were increased, whereas M2b-like and M2c-like subsets were decreased. Expression of CD273 and CD274 were reduced on M1-like cells, and CD273 expression was significantly decreased on M1-like and M2a-like subsets only, with non-significant trends in M2b-like cells. Non-responders to corticosteroid therapy demonstrated higher M1 polarization, increased M2a levels, reduced M2b and M2c subsets, and lower CD273 expression among M1 and M2a compared with responders. Significant correlations were identified between macrophage-like subsets and platelet count, hemoglobin, MPV, and PDW.

**Conclusion:**

These findings suggested that a pro-inflammatory M1-like polarization shift combined with an impaired dual PD-L1/PD-L2 checkpoint expression on M2b-like macrophages are significantly correlated with platelet indices and collectively contribute to ITP immunopathogenesis and treatment response, supporting macrophage polarization as a promising immunomodulatory target.

**Supplementary Information:**

The online version contains supplementary material available at https://doi.org/10.1007/s44313-026-00163-0.

## Introduction

Immune thrombocytopenic purpura (ITP) is a common disease characterized by reduced platelet count (PLT; < 100 × 10⁹/L) in the peripheral blood due to cell- and humoral-mediated destruction of one's own platelets. This process takes place in the spleen following platelets opsonization with autoantibodies and their phagocytosis by macrophages [1–3].

Macrophages play multiple roles in both innate and adaptive immune system. They engulf platelets with mediation by autoantibodies. Furthermore, they could polarize into classically activated M1 (Pro-inflammatory) macrophages or alternatively activated M2 (Anti-inflammatory) macrophages [4].

M1-like macrophages are considered proinflammatory and pivotal for inflammatory and autoimmune responses [5]. Alternatively activated M2-like macrophages are considered anti-inflammatory [6]. Macrophage polarization is regulated by the activation of several interrelated cellular signaling pathways [7].

In ITP, M1-like shows a preferential tendency at disease onset, and the M1/M2 balance recovers when remission occurs. When macrophages polarize to M1-like predominance, they also drive immune modulation of CD4 + T cells. Several therapies can target M1-like macrophages to restore the M1/M2 balance [4].

Programmed cell death protein (PD-1) is a transmembrane glycoprotein comprising an immunoreceptor tyrosine‐based inhibitory motif [8], which is mainly expressed on T cells in both CD4 and CD8 compartments [9]. The PD-1 binds with its two ligands CD274 (PD-L1) and CD273 (PD-L2), to turn off autoreactive T cells and induce peripheral tolerance [10]. PD-L1 is expressed on macrophages, cytotoxic T cells, and natural killer (NK) cells [11], while PD-L2, is expressed on macrophages and dendritic cells (DCs). Therefore, PD-L2 has an important role in the immunoregulatory effects of alternatively activated macrophages [12].

In ITP, there is an alteration in PD-1 and PD-L1 levels, that may play a role in its pathogenesis [13]. Previous studies have suggested a role for both macrophage polarization imbalance and PD-1/PD-L pathway dysregulation in ITP; however, the relationship between these immune alterations in children with acute ITP remains unclear. Therefore, we aimed to assess circulating monocyte/macrophage-like subsets, macrophage polarization, and PD-L1/PD-L2 expression in pediatric acute ITP.

## Patients and methods

This case–control study was conducted at Assiut University Children's Hospital. A total of 53 children were enrolled and divided into two groups: 32 patients with acute ITP and 21 age- and sex-matched healthy children as the control group. Eligible participants were children aged 1 to 18 years, who were newly diagnosed with acute ITP and planned to receive first-line corticosteroid therapy per institutional standard guidelines. All patients included in the study fulfilled the diagnostic criteria for primary ITP after thorough clinical and laboratory evaluations. The diagnosis was established based on the presence of isolated thrombocytopenia and typical clinical presentation, such as sudden onset of petechiae, purpura, and/or mucocutaneous bleeding in an apparently healthy child. Complete blood count (CBC) and peripheral blood smear were performed for all patients, in addition to bone marrow examination if clinically indicated. Although splenomegaly is uncommon in primary ITP, mild splenic enlargement was detected in some patients. Therefore, all patients especially those with splenomegaly underwent careful assessment to exclude secondary causes of thrombocytopenia. This evaluation included autoimmune profile assessment and viral screening for hepatitis B, hepatitis C, and human immunodeficiency virus (HIV). Children with identifiable causes of secondary ITP, including leukemia, aplastic anemia, congenital thrombocytopenia, systemic lupus erythematosus, or chronic infections such as HIV or hepatitis B or C virus or other chronic autoimmune, inflammatory, or malignant disease were excluded from the study. All patients with ITP received standard first-line therapy with oral prednisone (2–4 mg/kg/day; max 120 mg/day) for 5–7 days according to the American Society of Hematology [14]. Blood samples were collected at the time of diagnosis before initiating corticosteroid treatment.

### Clinical and laboratory assessment

All patients underwent a detailed medical history and thorough physical examination. The extent of bleeding was assessed using the Buchanan and Adix bleeding score [15]. The following laboratory tests were performed for all participants: CBC and flow cytometric assessment of circulating monocyte/macrophage-like subsets and their expression of PD-L1 and PD-L2. Patients were followed to assess treatment response based on the International Consensus Terminology and Criteria for Immune Thrombocytopenia for a Clinical Pediatric Population [1].

### Flow cytometric assessment of circulating macrophage subsets and their expression of PD-L1 and PD-L2

**Fig. 1:**
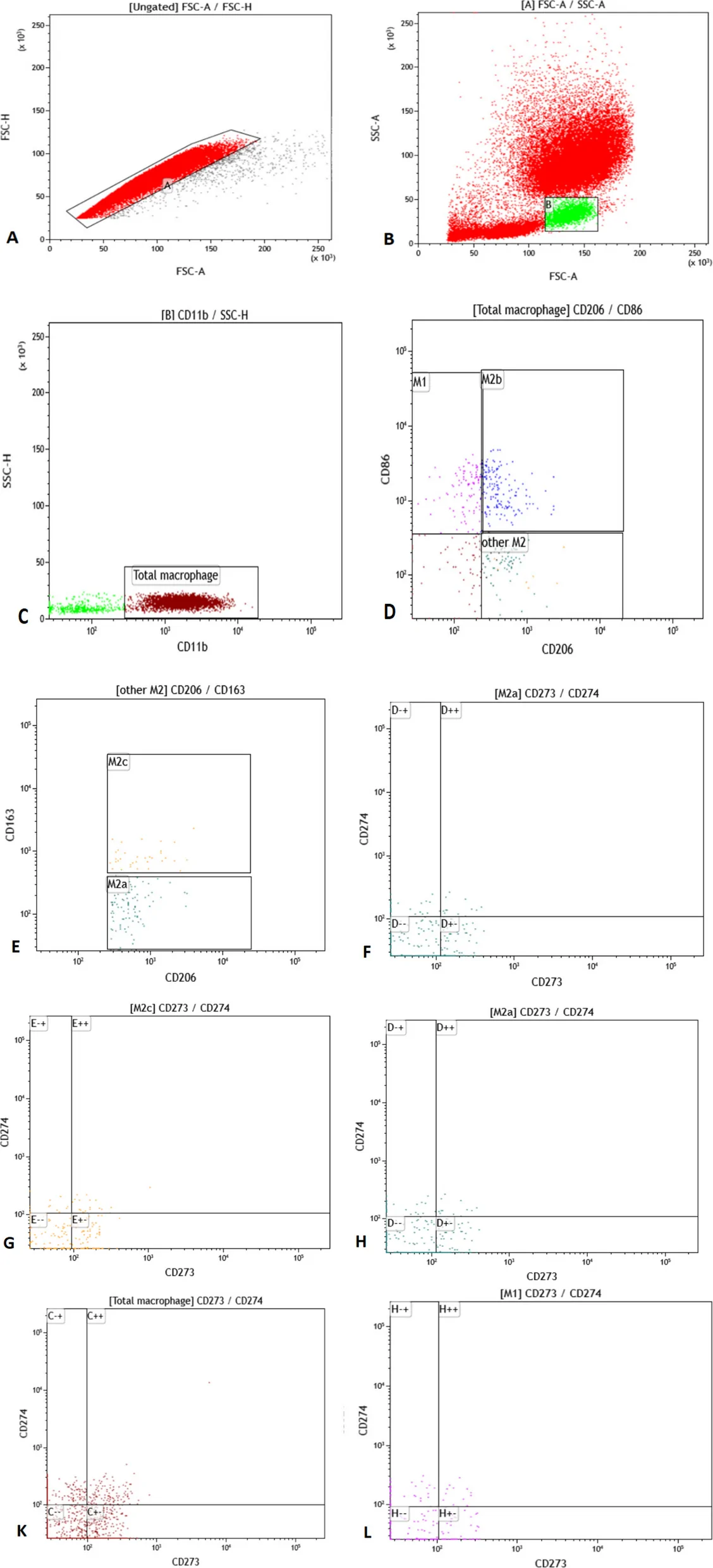
Flow cytometric assessment of circulating macrophage subsets and their expression of PD-L1 and PD-L2. **A**: Forward scatter area and height histogram was used to define the singlet population. **B**: Forward and side scatter histogram was used to define the monocytes/macrophage population (**B**). **C**: CD11b was assessed within the monocytes/macrophage population to detect total macrophage (CD11b + cells). **D-E**: The expression of CD206, CD86, and CD163 were then assessed within the total macrophage for further identification of macrophage subsets as follow: CD86 + CD11b + M1, CD86 + CD206 + CD11b + M2b, CD206 + CD11b + CD86-CD163- M2a, CD86-CD163 + CD206 + CD11b + M2c. **F-L**: PD-L1 (CD274) and PD-L2 (CD273) expression were assessed among different macrophage subsets

Monocyte-macrophage polarization tendencies were assessed by flow cytometry using 10 µl of each of APCH7 conjugated anti-CD274 (PD-L1), APC conjugated anti-CD273 (PD-L2), FITC conjugated anti-CD11b, V450 conjugated anti-CD206, PE conjugated anti-CD86 and Per-Cp conjugated anti-CD163, which were incubated with 100 µl of blood samples for 15 min at 4 °C in the dark. After RBCs lysis, washing with phosphate-buffered saline (PBS) was performed, then the RBCs were resuspended in PBS. Flow cytometric analysis was done by FACSCanto flow cytometry using FACSDIVA 7.0 software (Becton Dickinson, USA). A minimum of 50,000 events was collected for each sample analysis. A forward and side scatter histogram was used to define the monocyte population, then followed by the detection of total CD11b + monocyte-macrophage-like cells. Next, M1-like (CD86⁺CD11b⁺), M2a-like (CD206⁺CD11b⁺CD86⁻CD163⁻), M2b-like (CD86⁺CD206⁺CD11b⁺), and M2c-like (CD86⁻CD163⁺CD206⁺CD11b⁺) monocyte/macrophage-like subsets were defined based on surface marker expression. Lastly, PD-L1 (CD274) and PD-L2 (CD273) expression were assessed across these phenotypically defined subsets. The strategy of monocyte-macrophage subset gating is demonstrated in Fig. 1.

### Statistical analysis

All data were analyzed using R software version 4.3.0 (R Foundation for Statistical Computing, Vienna, Austria). The hematological indices and flow cytometry data of the macrophage subtypes were initially assessed for normality using the Shapiro–Wilk test. Data was expressed as the median and interquartile range (IQR) because they failed to satisfy the assumption of normality, and consequently subjected to the Mann–Whitney U-test to compare cases and controls. The Spearman correlation test was performed to assess correlations.

## Results

### Peripheral hemogram indices of patients with ITP in comparison to healthy controls

The clinical and demographic data of patients with ITP and healthy controls are shown in Table 1. Table 2 shows the hematological data of patients with ITP and healthy controls. It revealed a significant difference in the medians of the hematological indices between patients and controls. The patients with ITP included in the study had significantly reduced hemoglobin (Hb) levels, PLT, and lymphocytic count, along with increased mean platelet volume (MPV), and platelet distribution width (PDW) compared with healthy controls.

**Table 1:** Clinical and demographic data of patients and healthy controls

Characteristic	Cases (*n* = 32)	Controls (*n* = 21)	*P* value
Sex, n (%)			
Female	15 (47%)	11 (52.4%)	0.78
Male	17 (53%)	10 (47.6%)	
Age (years), mean ± SD	6 ± 3 years	7 ± 4 years	0.26
Skin and soft tissue manifestation (petechiae, skin bruises, mucosal bleeding)			
Petechiae	27 (84.4%)	-	-
Skin bruises	23 (71.9%)	-	-
Mucosal bleeding	17 (54.1%)	-	-
Splenomegaly			
Yes	5 (15.6%)	-	-
No	27 (84.4%)	-	-
Buchanan and Adix bleeding score grade (0, 1, 2, 3, 4, 5)			
Grade 0	3 (9.4%)	-	-
Grade 1	4 (12.5%)	-	-
Grade 2	8 (25%)	-	-
Grade 3	11 (34.3%)	-	-
Grade 4–5	6 (18.8%)	-	-
Response to treatment			
No response	19 (59.4%)	-	-
Partial response	9 (28.1%)	-	-
Complete response	4 (12.5%)	-	-

Response categories (no response, partial response, complete response) were defined according to the International Consensus Terminology and Criteria for Immune Thrombocytopenia for a Clinical Pediatric Population [1]

**Table 2:** Hematological data of patients with ITP and healthy controls

CBC elements	Group	Median [IQR]	*P* value
Hb (g/L)	Patients	10.75 (10.25—11.30)	< 0.001
	Control	12.30 (11.70—12.80)	
WBCs	Patients	6.05 (3.80—7.85)	0.23
	Control	7.00 (5.50—8.60)	
Platelets	Patients	30 (23—43)	< 0.001
	Controls	312 (279—354)	
MPV	Patients	12.0 (9.3—15.3)	< 0.001
	Controls	8.0 (7.3—8.9)	
PDW	Patients	15.5 (13.0—18.5)	0.014
	Controls	13.3 (11.6—14.4)	
Neutrophils	Patients	3.13 (1.79—5.07)	0.93
	Controls	2.92 (2.55—4.69)	
Lymphocyte (count)	Patients	2.00 (1.47—2.59)	0.003
	Controls	2.82 (2.37—3.37)	
Lymphocyte (%)	Patients	40 (27—50)	0.35
	Controls	41 (33—47)	
Monocytes	Patients	0.37 (0.33—0.54)	0.64
	Controls	0.46 (0.32—0.61)	

*CBC* Complete blood count, *IQR* Interquartile range, *Hb *Hemoglobin, *WBCs* White blood cells, *MPV* Mean platelet volume, and *PDW *platelet distribution width

Mann–Whitney U-test, *P* value was significant at < 0.05

### Enhanced divergence of monocyte-macrophage polarization towards M1-like cells

**Fig. 2:**
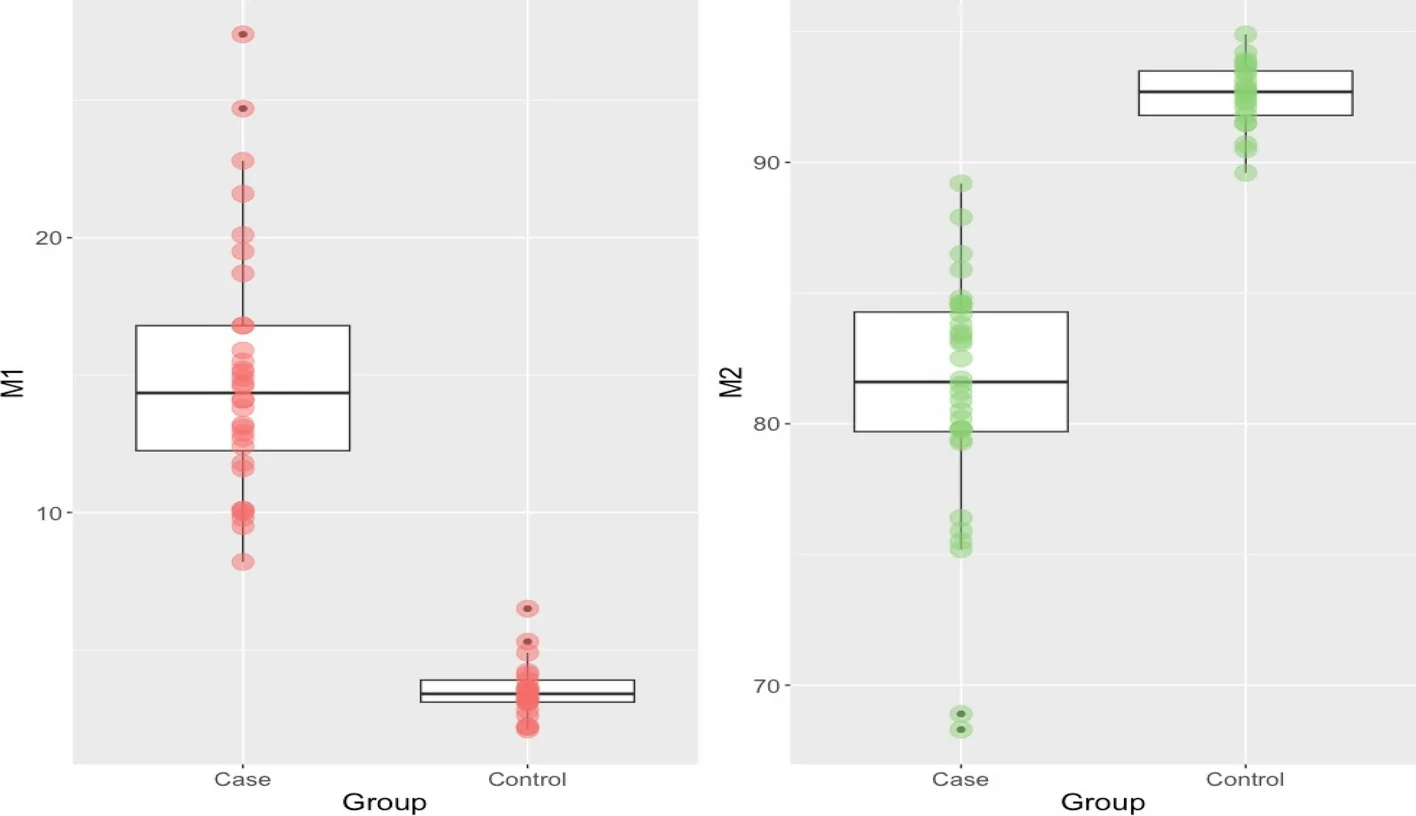
Differences in percentages of monocyte-macrophage phenotypes between ITP cases and controls. Box-and-whisker plots show the percentages of total CD11b⁺ monocyte-macrophage-like cells, M1-like, and total M2-like subsets in patients with ITP (*n* = 32) vs. healthy controls (*n* = 21), gated as described in Fig. 1. Boxes represent the median and interquartile range; whiskers denote the range. Statistical comparisons were performed using the Mann–Whitney U-test

We detected an elevated polarization of M1-like monocyte-macrophage in the patients group compared with the control group, which was clearly expressed by the percentages of the flowcytometric analysis of M1 subtype (median [IQR]: 14.4% [12.1—16.8%] vs. 3.4% [3.1—3.9%], respectively, *P* < 0.001). On the contrary, M2-like monocyte-macrophage prevalence was less evident in ITP with a median [IQR] of 81.6% [79.6—84.4%] in patients vs. 92.7% [91.8—93.5%] in controls (*P* < 0.001) (Table 3) (Fig. 2).

**Table 3:** Monocyte-macrophage subtypes polarization difference between patients with ITP and controls

(%)	Group	Median [IQR]	*P* value
Total monocytes	Patients	6.40 (5.00—7.85)	0.5
	Controls	5.70 (5.00—6.80)	
Total CD11b + monocyte-macrophage-like cells	Patients	79 (69—83)	0.04
	Controls	71 (70—74)	
M1-like monocyte-macrophage	Patients	14.4 (12.1—16.8)	< 0.001
	Controls	3.4 (3.1—3.9)	
M2-like monocyte-macrophage	Patients	81.6 (79.6—84.4)	< 0.001
	Controls	92.7 (91.8—93.5)	
M2a-like monocyte-macrophage	Patients	58 (54—67)	< 0.001
	Controls	29 (23—32)	
M2b-like monocyte-macrophage	Patients	11 (5—14)	< 0.001
	Controls	24 (18—28)	
M2c-like monocyte-macrophage	Patients	7 (5—11)	< 0.001
	Controls	38 (34—48)	

Mann–Whitney U-test, *P* value was significant at < 0.05

### Altered M2-like monocyte-macrophage subtypes polarization in patients relative to controls

Recently, M2-like monocyte-macrophage have garnered considerable attention due to their unique plasticity, complex cytokine profiles, and multifaceted functions, which led to their categorization into several subsets, with the most studied being M2a, M2b, and M2c.

Regarding the M2-like monocyte-macrophage subpopulation, our data showed that there was an increased abundance of CD206 + CD163- CD86- M2a-like monocyte-macrophage subtype in the ITP group in comparison to the control group, demonstrated by increased percentages in patients 58% (54 – 67%) vs. 29% (23 – 32%) in controls (*P* < 0.001).

**Fig. 3:**
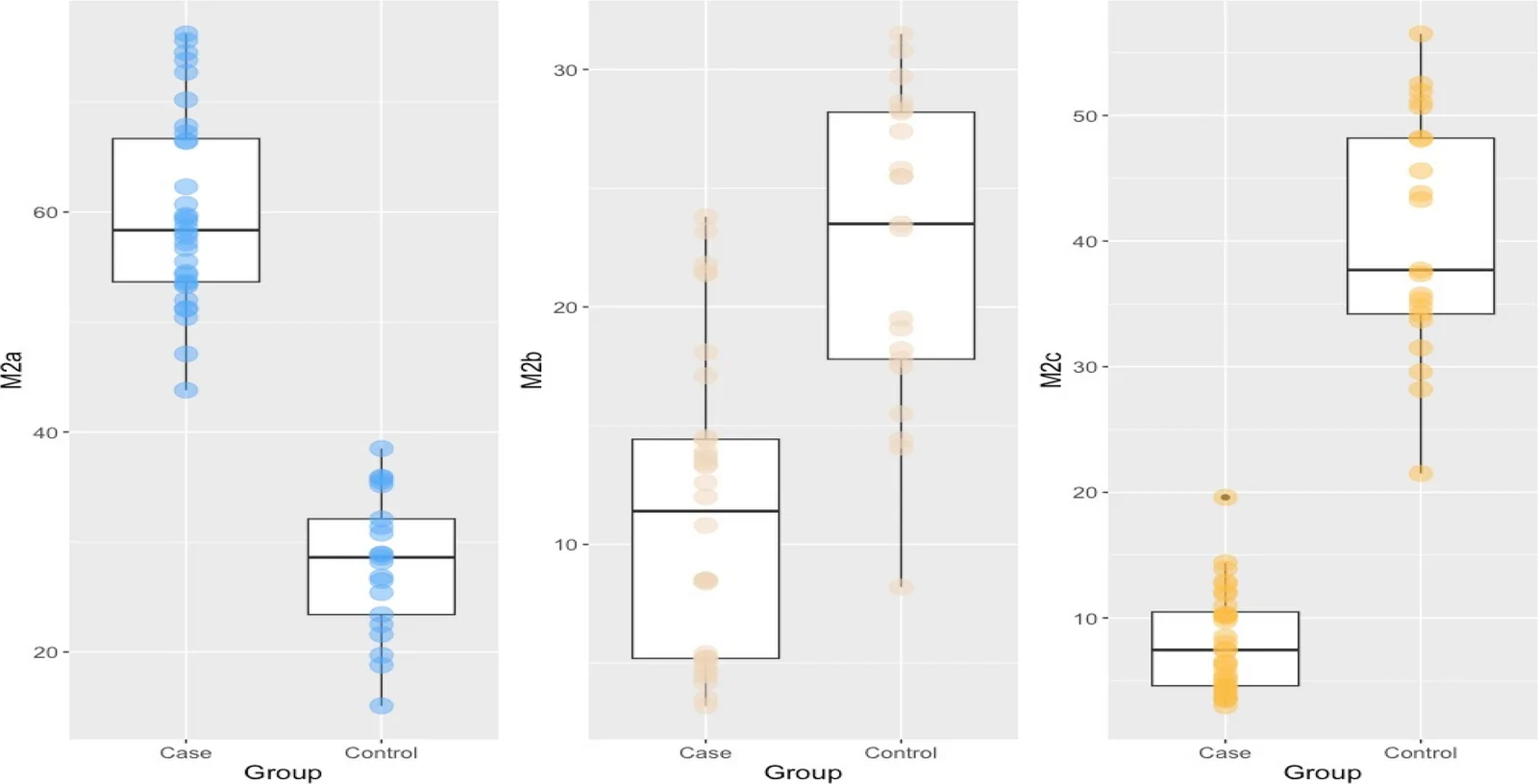
Differences in percentages of M2-like monocyte-macrophage subsets between ITP cases and controls. Box-and-whisker plots show the percentages of M2a-like, M2b-like, and M2c-like monocyte-macrophage subsets, gated as CD206⁺CD11b⁺CD86⁻CD163⁻, CD86⁺CD206⁺CD11b⁺, and CD86⁻CD163⁺CD206⁺CD11b⁺ cells respectively, in patients with ITP (*n* = 32) vs. healthy controls (*n* = 21). Boxes represent the median and interquartile range; whiskers denote the range. Statistical comparisons were performed using the Mann–Whitney U-test

On the other hand, CD86 + CD206 + M2b-like monocyte-macrophage and CD206 + CD163 + M2c-like monocyte-macrophage percentages were significantly less prominent in patients with ITP vs controls (11% [5 – 14%] vs. 24% [18 – 28%], *P* < 0.001 and 7% [5 – 11%] vs. 38% [34 – 48%], *P* < 0.001, respectively) (Table 3) (Fig. 3).

### Differential expression of CD273 (PD-L2) and CD274 (PD-L1) on monocyte-macrophage subsets between patients with ITP and controls

**Fig. 4:**
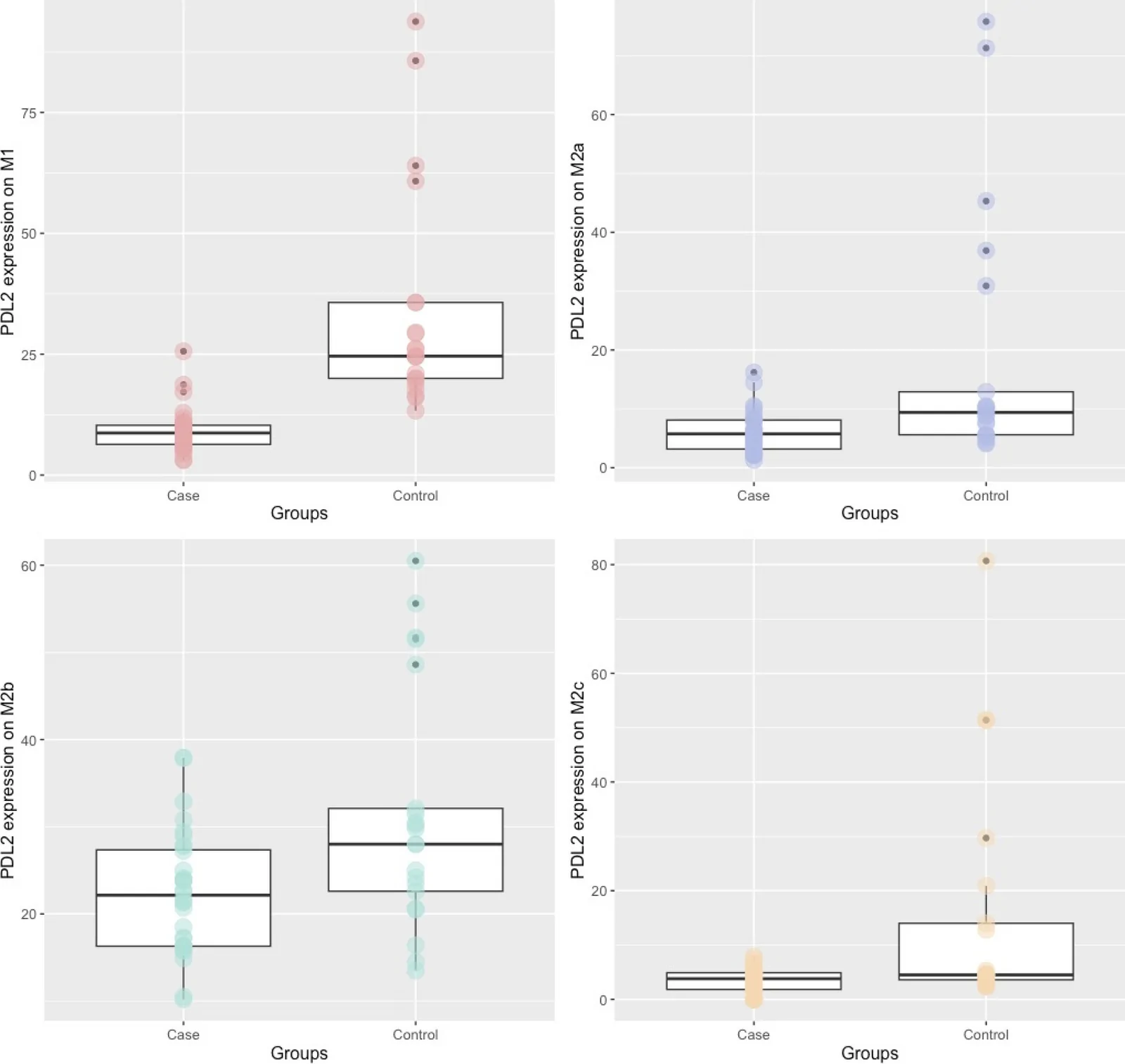
Differences in percentages of PD-L2 (CD273) expressed on monocyte-macrophage phenotypes between ITP cases and controls. Box-and-whisker plots show the percentage of CD273⁺ (PD-L2⁺) cells within the M1-like, M2a-like, M2b-like, and M2c-like monocyte-macrophage subsets in patients with ITP (*n* = 32) vs. healthy controls (*n* = 21). Boxes represent the median and interquartile range; whiskers denote the range. Statistical comparisons were performed using the Mann–Whitney U-test

There were significantly reduced levels of expression of both CD273 (PD-L2) and CD274 (PD-L1) on M1-like monocyte-macrophage in patients with ITP compared with controls; median [IQR]: 9 [6–10] vs. 25 [20–36] (*P* < 0.001) and 3.9 [0.9—6.2] vs. 6.7 [3.3—10.7] (*P* = 0.02) for CD273 and CD274, respectively (Table 4) (Fig. 4).

**Table 4:** Expression of PD-L1 (CD274) and PD-L2 (CD273) on the surface of different monocyte-macrophage subtypes in patients with ITP and controls

(%)	Group	Median [IQR]	*P* value
CD273 + Total monocyte-macrophage	Patients	3 (1—10)	0.003
	Controls	7 (6—9)	
CD274 + Total monocyte-macrophage	Patients	1.20 (0.90—2.20)	0.83
	Controls	1.10 (0.80—2.10)	
CD273 + M1-like monocyte-macrophage	Patients	9 (6—10)	< 0.001
	Controls	25 (20—36)	
CD274 + M1-like monocyte-macrophage	Patients	3.9 (0.9—6.2)	0.02
	Controls	6.7 (3.3—10.7)	
CD273 + M2a-like monocyte-macrophage	Patients	6 (3—8)	0.001
	Controls	9 (6—13)	
CD274 + M2a-like monocyte-macrophage	Patients	1.50 (0.70—2.25)	0.7
	Controls	1.40 (0.90—1.70)	
CD273 + M2b-like monocyte-macrophage	Patients	22 (16—28)	0.02
	Controls	28 (23—32)	
CD274 + M2b-like monocyte-macrophage	Patients	5.3 (3.5—7.4)	0.6
	Controls	4.4 (4.0—5.0)	
CD273 + M2c-like monocyte-macrophage	Patients	4 (2—5)	0.03
	Controls	5 (4—14)	
CD274 + M2c-like monocyte-macrophage	Patients	1.05 (0.00—3.10)	0.74
	Controls	1.00 (0.70—1.40)	

Mann–Whitney U-test, *P* value was significant at < 0.05

There was also a significant decline in the median percentages of CD273 (PD-L2) expression on cell surfaces of all M2-like subpopulations.

For CD273 expression on M2a-like subtype: the median [IQR] was 6 [3–8] vs. 25 [20–36] (*P* = 0.001); on M2b-like subtype: the median [IQR] was 22 [16–28] vs. 28 [23–32] (*P* = 0.02); and on M2c-like subtype: the median [IQR] was 4 [2–5] vs. 5 [4–14] (*P* = 0.03) for patients with ITP vs. healthy controls, respectively.

Furthermore, there was a tendency towards an elevation in CD274 (PD-L1) expression on all M2-like subtypes. However, this difference didn’t reach statistical significance.

### Relation of monocyte-macrophage phenotype and their expression of CD273 and CD274 in patients with ITP in response to corticosteroid treatment

Mann–Whitney U test was conducted to examine the relation of monocyte-macrophage phenotypes in response of patients with ITP to treatment (No response, response, complete response), which was categorized into a binary outcome (Complete or partial response/No response). Nineteen patients showed no response, while 13 showed partial to complete response.

Elevated polarization of M1-like monocyte-macrophage was detected in the non-responder patients with ITP compared with the responders. Furthermore, higher levels of M2a and lower levels of M2b and M2c were observed in the non-responders group. CD273⁺ M1 (*P* = 0.01) and CD273⁺ M2a (*P* = 0.04) were significantly lower in the non-responders; CD273⁺ M2b showed a non-significant trend (*P* = 0.074), and CD273⁺ M2c was essentially identical between groups (3.3 vs. 3.6, *P* = 0.91). The results revealing a significant association of various monocyte-macrophage phenotypes with treatment response are demonstrated in Table 5.

**Table 5:** Relation of monocyte-macrophage subset polarization and their expression of CD273 and CD274 with corticosteroid treatment response in patients with ITP

(%)	Group	N	Median [IQR]	*P* value
Total CD11b + monocyte-macrophage-like cells	No response	19	82 (78—86)	0.02
	Partial or complete response	13	72 (56—80)	
CD273 + CD11b + monocyte-macrophage-like cells	No response	19	1.90 (1.20—9.80)	0.6
	Partial or complete response	13	4.30 (1.20—9.70)	
CD274 + CD11b + monocyte-macrophage-like cells	No response	19	1.20 (0.90—1.60)	0.7
	Partial or complete response	13	1.20 (0.90—2.30)	
M1-likemonocyte-macrophage	No response	19	15.2 (13.8—20.1)	0.013
	Partial or complete response	13	12.9 (11.6—14.1)	
CD273 + M1-likemonocyte-macrophage	No response	19	6.4 (5.5—10.1)	0.01
	Partial or complete response	13	10.1 (9.5—11.8)	
CD274 + M1-likemonocyte-macrophage	No response	19	4.00 (0.00—6.00)	0.33
	Partial or complete response	13	3.80 (3.00—9.30)	
M2-likemonocyte-macrophage	No response	19	79.8 (75.9—81.7)	< 0.001
	Partial or complete response	13	84.5 (83.1—85.9)	
M2a-likemonocyte-macrophage	No response	19	60 (56—73)	0.032
	Partial or complete response	13	55 (51—60)	
CD273 + M2a-likemonocyte-macrophage	No response	19	4.3 (2.3—7.8)	0.04
	Partial or complete response	13	6.3 (5.6—8.1)	
CD274 + M2a-likemonocyte-macrophage	No response	19	1.30 (0.80—1.90)	0.34
	Partial or complete response	13	1.80 (0.60—2.90)	
M2b-likemonocyte- macrophage	No response	19	8.4 (4.5—13.7)	0.014
	Partial or complete response	13	13.6 (8.5—21.4)	
CD273 + M2b-likemonocyte-macrophage	No response	19	21 (16—24)	0.074
	Partial or complete response	13	24 (19—31)	
CD274 + M2b-likemonocyte-macrophages	No response	19	4.2 (3.4—7.1)	0.12
	Partial or complete response	13	6.9 (4.4—13.2)	
M2c-likemonocyte-macrophage	No response	19	5.1 (4.0—7.4)	< 0.001
	Partial or complete response	13	10.3 (9.8—12.9)	
CD273 + M2c-likemonocyte-macrophage	No response	19	4.30 (0.00—4.90)	0.91
	Partial or complete response	13	3.70 (2.00—4.60)	
CD274 + M2c-likemonocyte-macrophage	No response	19	1.30 (0.00—3.70)	0.55
	Partial or complete response	13	0.70 (0.00—2.80)	

Mann–Whitney U-test, *P* value was significant at < 0.05

### Correlation between hematological parameters and monocyte-macrophages subtypes

Upon analyzing the correlation between the most vital parameters of hemogram of patients with ITP, including PLT, Hb, PDW, and MPV, and the different monocyte-macrophage subsets and their expression of CD273 and CD274, it was found that there were significant correlations between various monocyte-macrophage subtypes and some hematological parameters, as shown in Table 6 (condensed) and Supplementary Tables S1 and S2. The PLT showed a positive correlation with total M2 like macrophages and negative correlations with MPV and PDW.

**Table 6:** Key significant correlations between hematological parameters and monocyte–macrophage subsets in patients with ITP. Full correlation matrices are provided in Supplementary Tables S1 and S2

Variable 1	Variable 2	Spearman ρ	*P* value
Platelet count	MPV	− 0.76	< 0.001
Platelet count	Hb	0.65	< 0.001
Platelet count	PDW	− 0.56	< 0.001
MPV	PDW	0.65	< 0.001
MPV	Hb	− 0.50	0.003
M1-like	PDW	0.49	0.004
M1-like	M2-like (total)	− 0.87	< 0.001
M2b-like	M2a-like	− 0.88	< 0.001
CD274⁺ M2b	Platelet count	0.70	< 0.001
CD274⁺ M2b	MPV	− 0.60	< 0.001
CD274⁺ M2b	PDW	− 0.55	0.001
CD273⁺ M2b	Platelet count	0.64	< 0.001
CD273⁺ M2b	PDW	− 0.53	0.002
CD273⁺ M2b	MPV	− 0.46	0.01

Spearman correlation coefficient (ρ); *MPV* mean platelet volume; *Hb* hemoglobin; *PDW* platelet distribution width; *CD273* PD-L2, *CD274* PD-L1. Only correlations with |ρ|≥ 0.40 and *p* < 0.05 are shown

CD274⁺ M2b like cells were strongly positively correlated with PLT and Hb level, while showing negative correlations with MPV and PDW. Similarly, CD273⁺ M2b like cells showed a strong positive correlation with PLT and negative correlations with MPV and PDW. In contrast, CD274⁺ M1 macrophages demonstrated only a modest positive correlation with PLT. These findings suggested that M2b macrophage polarization, particularly CD273/CD274-expressing subsets, is associated with more favorable hematological profiles in patients with ITP.

## Discussion

ITP is an acquired autoimmune disease involving increased platelet destruction and impaired bone marrow synthesis, which is driven by autoantibody-mediated phagocytosis by macrophages [1]. The pathogenesis of ITP is multifactorial; it is linked to T-cell dysfunction, dysregulated immune response, and abnormal activity of cytotoxic T-cell, as well as macrophage abnormalities, which plays an essential role. Macrophages are important antigen-presenting cells, classified into pro-inflammatory (M1-like) or anti-inflammatory (M2-like) categories in response to cytokines and transcription factors [2, 3].

The present study showed a distinct imbalance in monocyte-macrophage polarization between children with ITP and healthy controls, with patients exhibiting a predominance of proinflammatory M1-likemonocyte-macrophage and reduced levels of M2-likemonocyte-macrophage. The predominance of the M1-like phenotype likely reflects ongoing immune activation. This proinflammatory state may not only enhance platelet destruction but also interfere with megakaryocyte maturation, thereby linking monocyte-macrophage imbalance directly to the development of thrombocytopenia [16, 17].

In contrast, M2-like monocyte-macrophages were found to be more abundant in healthy controls. These results are consistent with previous studies. For instance, Hong-Yan Zhao et al. recognized an increase in M1 and a reduction in M2 polarization in bone marrow derived macrophages of patients who developed post-transplant thrombocytopenia [16]. Similarly, Mengzhu Shen et al. verified that the knocking out PI3K in mouse bone marrow macrophages delayed platelet synthesis, and showed that patients with ITP exhibited an altered M1 and M2 ratio with reduced PI3K/AKT signaling, suggesting that M2 macrophages facilitate megakaryocyte differentiation [17].

Moreover, numerous prior studies highlighted the vital role of the PI3K/AKT pathway in inhibiting M1-like macrophage polarization and encouraging the anti-inflammatory M2 phenotype by suppressing TLR/NF-κB and enhancing STAT3 signalling [ 7, 7, 18]. Also, PI3K/AKT pathway modulates autophagy, which guards against apoptosis in several disease conditions, including ITP [18]. In ITP, decreased platelet autophagy leads to augmented apoptosis, resulting in autoimmune platelet destruction [19]. Furthermore, mutations affecting PI3K/AKT pathway signaling can disturb platelet autophagy, increase T-cell autoimmunity, and encourage M1 polarization [20], additionally disrupting megakaryocyte maturation and differentiation, thus worsening ITP.

The results of the present study showed a predominance of M1-like monocyte-macrophage polarization in patients with ITP relative to healthy controls. However, variability was observed between M2 subtypes: we reported an elevated M2a-like monocyte-macrophage subtype in patients with ITP, while M2b and M2c were less predominant compared with healthy controls. The increase of the M2a-like subset in our studied patients likely reflected a compensatory yet ineffective response to ongoing inflammation. Although M2a macrophages are involved in tissue repair and fibrosis, they lack the strong immunosuppressive capacity characteristic of M2c macrophages. Therefore, the associated reduction in M2c-like cells, which are critical for IL-10–mediated immune tolerance, suggests that regulatory macrophage functions are selectively impaired in ITP [21, 22]. This imbalance may permit continued autoreactive T-cell activation and platelet destruction despite the presence of alternative activation signals [23]. Overall, changes in M2 macrophage subtypes indicates functional tilting toward tissue remodeling rather than true immunoregulation, thereby contributing to persistent immune dysregulation in ITP.

The anti-inflammatory and immunoregulatory roles of M2-like macrophages and their subtypes in various diseases have been explored previously in several studies. However, their exact contributions in ITP remain poorly understood [24]. The most studied subtype was M2a-like, which promotes tissue repair, cell proliferation, and fibrosis [25–27]. Additionally, J. Zhang et al. demonstrated its role in axonal regeneration via Arg-1 production [28]. Nonetheless, elevated M2a-like levels have also been linked to the deterioration of some disease conditions, like asthma and intervertebral disc degeneration, through pro-inflammatory pathways such as IL-13Rα2/MAPK signaling [5]. Moreover, excessive M2a-like activity may contribute to kidney dysfunction in lupus nephritis [29].

On the other hand, M2b-like macrophages display both pro- and anti-inflammatory effects [30]. They have neuroprotective roles in Alzheimer’s and spinal cord injury models through IL-10 secretion [31, 32], and endorse recovery in cardiovascular disease models [33]. However, their immunosuppressive effects can impair the elimination of pathogens, contributing to autoimmune disorders such as inflammatory bowel disease and systemic juvenile idiopathic arthritis [34]. M2c-like is a subtype of M2 macrophage, which has the most anti-inflammatory effects, and is involved in immune tolerance and tissue repair [35]. Studies showed that M2c-like cells, characterized by high IL-10 expression, more effectively reduce inflammation and fibrosis in lung injury and kidney disease than other M2-like cells [35, 36]. This supports our finding of higher M2a-like levels together with reduced M2c and M2b subtypes expression in patients with ITP. PD-1 is a transmembrane glycoprotein that’s mainly expressed on macrophages, cytotoxic T cells, B cells, DCs, regulatory T cells, and NK cells. It is also expressed on hematopoietic and non-hematopoietic cells [11]. PD-1 has two identified ligands PD-L1 (CD274) and PD-L2 (CD273). We observed differences in PD-L1 and PD-L2 expression across monocyte-macrophage subtypes.

Patients with ITP showed reduced PD-L1 and PD-L2 on M1-like monocyte-macrophage, whereas M2-like subtypes had decreased levels of PD-L2 only and PD-L1 expression was increased on the cell surface of all M2a, M2b, and M2c macrophage subtypes. However, only PD-L2 yielded a significant difference. PD-1 interaction with PD-L1 and PD-L2 can deactivate autoreactive T cells and induce peripheral tolerance [10].

Our findings are consistent with previous studies reporting increased PD-1 expression on both CD4 + and CD8 + T cells from patients with ITP compared with healthy controls' T cells as well as reduced PD-L1 on DCs in patients with active ITP [37, 38].

Previous studies have indicated that while PD-L1 can be readily induced on the surfaces of various cell types, the expression of PD-L2 is much more restricted and can be strongly upregulated only by IL-4 or a Th2 response in macrophages [39, 40]. This observation may explain our findings, which propose that there is a higher expression of PD-L2 across all M2-like monocyte-macrophage subpopulations in healthy controls when compared to patients with ITP. Both PD-L1 and PD-L2 on M2b-like macrophages may constitute a dual tolerogenic checkpoint axis in pediatric ITP, as suggested by the significant reduction of PD-L2 across all M2 subsets and the strong correlation of CD274⁺ (PD-L1) on M2b with PLT (ρ = 0.70) and platelet indices. This regulatory pathway appears to be disturbed in ITP, and functional studies are needed to determine the relative contributions of each ligand.

The current study found a significant association between monocyte-macrophage subsets and treatment response in patients with ITP. Patients who showed no response to therapy tended to have higher levels of M1 and M2a-like monocyte-macrophage subsets, whereas those who achieved a partial or complete response had increased levels of the M2b and M2c-like subtype. These findings suggest that a shift toward M2b and M2c-like polarization may be linked to a better treatment response; however, further confirmation is needed. Our findings may also have practical implications for managing ITP. Therapies that can shift macrophage activity away from the proinflammatory M1 or M2a-like types and toward the more regulatory M2c-like subtype could help restore immune balance and improve response to treatment. In addition, approaches that modulate macrophage function, either directly or through pathways such as PI3K/AKT, may represent promising future strategies to support platelet recovery and better disease control. Future studies that examine macrophage polarization in bone marrow samples or at different stages of the disease could provide deeper insight into how these subsets influence ITP progression and response to treatment. Our findings are consistent with those of Q. Feng et al., who examined the effect of high-dose steroids or all-trans retinoic acid (ATRA) on the expression of M1 and M2 markers in 62 patients with ITP. They reported that the treatment reduced the M1/M2 ratio, shifting toward the anti-inflammatory M2-like phenotype, which is essential for restoring immune balance [41]. In their study, M1 markers such as CD80, CD86, and CCR7 were decreased, while M2c markers, including CD163 and CD209, were increased, with no significant change in the M2a marker CD206. Collectively, these results highlight the important role of M2c-like macrophages in modulating the response to treatment [41]. Furthermore, expression of the immune checkpoint ligand CD273 (PD-L2) was significantly lower in non-responders for M1-like (*P* = 0.01) and M2a-like (*P* = 0.04) subsets; whereas, CD273 on M2b-like showed a non-significant trend (*P* = 0.074), and CD273 on M2c-like did not differ between groups (*P* = 0.91). These findings further highlighted a subset-specific defect in inhibitory immune signaling as opposed to a uniform across-all-subsets reduction. Reduced PD-L2 expression may limit macrophage-mediated T-cell regulation, thereby enhancing immune activation and platelet destruction despite corticosteroid therapy. Collectively, these findings support a significant association between monocyte–macrophage phenotype distribution, checkpoint molecule expression, and treatment response in ITP, underscoring the potential role of innate immune dysregulation in corticosteroid refractoriness. Our data is consistent with Shao et al., demonstrating that macrophage polarization plays a critical role in ITP pathogenesis and treatment response. In particular, prior studies have shown that low-dose decitabine has been shown to promote M2-like macrophage polarization in patients with ITP, increasing markers such as PPARγ and Arg-1 and enhancing IL-10 production, which correlates with improved immunoregulation compared to pro-inflammatory M1-like macrophages. This further supports the theory that skewing macrophage polarization toward an anti-inflammatory phenotype may improve clinical outcomes in refractory ITP, consistent with the higher M1-like prevalence observed in non-responders in our cohort [42]. Another study by Feng et al. have demonstrated not only a skewed M1/M2 balance in ITP patients but also that therapeutic interventions such as high-dose dexamethasone and ATRA restore macrophage polarization toward an M2-like phenotype. These agents reduce pro-inflammatory cytokines and costimulatory markers and augment IL-10 production, supporting enhanced immune tolerance. These findings align with our observation that responders to corticosteroids display a more pronounced anti-inflammatory phenotype, whereas non-responders have elevated M1 and reduced M2-like subsets [41]. 

Beyond phenotypic differences, monocyte–macrophage polarization patterns correlated significantly with key hematological parameters. M1-like macrophages showed a strong inverse correlation with M2-like cells (overall *P* < 0.001), consistent with a shift in polarization balance rather than independent subset expansion. Among checkpoint-expressing subsets, CD274⁺ (PD-L1⁺) M2b-like macrophages demonstrated the strongest association with PLT (*P* < 0.001) and inverse correlations with MPV (*P* < 0.001) and PDW (*P* = 0.001), indices that reflect abnormal platelet production kinetics. Similarly, CD273⁺ (PD-L2⁺) M2b-like cells correlated positively with PLT (*P* < 0.001) and negatively with MPV and PDW. These findings suggested that the M2b-like macrophage subset, through dual PD-L1/PD-L2 checkpoint expression, might be playing a central role in maintaining hematological stability in ITP, potentially by restraining autoreactive T-cell activity and reducing antibody-mediated platelet clearance. To our knowledge, this is the first study to demonstrate direct correlation between macrophage checkpoint molecule expression and platelet indices in pediatric ITP. Therefore, the observed associations warrant further investigation in larger prospective cohorts. Furthermore, pro-inflammatory M1-like macrophages correlated with adverse PDW (*P* = 0.004); however, they did not show a significant correlation with MPV (*P* = 0.3), indicating increased platelet destruction and turnover. These findings underscore the clinical relevance of macrophage induced immune dysregulation in shaping disease severity and therapeutic responsiveness.

The imbalance in monocyte-macrophage polarization observed in our study may help in clarifying the hematologic abnormalities associated with ITP. The dominance of proinflammatory M1 macrophages, along with the relative decline in anti-inflammatory M2 cells, likely contributes to both increased platelet destruction and insufficient platelet production. This disturbed immune balance corresponds with the low PLT and changes in platelet indices found in our patients, suggesting that macrophage-driven inflammation plays a key role in the disruption of normal thrombopoiesis.

## Conclusion

The current study highlights a pro-inflammatory shift in monocyte–macrophage polarization in pediatric acute ITP, characterized by M1-like and M2a-like predominance along with reduced M2b-like and M2c-like subsets. This imbalance was accompanied by subset-specific reductions in CD273 (PD-L2), which was most pronounced in M1-like and M2a-like cells. CD274 (PD-L1) expression on M2b-like macrophages did not differ significantly between patients with ITP and healthy controls; however, within the patient group, CD274⁺ M2b-like cells showed the strongest correlations with platelet count and platelet indices, linking this checkpoint to overall hematological stability rather than to an isolated expression defect. Enhanced M2c-like polarization associated with corticosteroid responsiveness further underscores the therapeutic relevance of macrophage phenotyping in ITP. These findings support macrophage polarization and PD-L1/PD-L2 checkpoint expression as promising immunomodulatory targets warranting further functional validation in larger prospective studies.

## Limitations

The present study had several limitations. First, this was a single-center study and a relatively small sample size, which may restrict the generalizability of findings. Secondly, no multiple statistical correlations were performed without adjustment for multiple comparisons. Thirdly, the cross-sectional design hindered the assessment of dynamic changes over time and did not allow evaluation of temporal changes, treatment-related alterations, or intra-individual variability in macrophage polarization and checkpoint expression over the disease course. Fourthly, the study investigated the peripheral blood monocyte-macrophages instead of those from the bone marrow. Lastly, macrophages were identified based on surface marker phenotyping only without functional assessment.

## Supplementary Information

Supplementary Material 1.

## Data Availability

All data generated or analyzed during this study are included in this article. Further inquiries can be directed to the corresponding author.
