# Supplementary material for: Unveiling the role of circulating monocyte-macrophage polarization state and PD-L1/PD-L2 expression in newly diagnosed pediatric ITP
Source: Blood Res. 2026 Oct 1;61(1):59. doi: 10.1007/s44313-026-00163-0 (PMC13631121; doi:10.1007/s44313-026-00163-0)
Supplement: Supplementary file 1 [file 44313_2026_163_MOESM1_ESM.docx]

**Supplementary table (1): Correlation between monocyte-macrophage subtypes and hematological parameters in ITP patients.**

| Variable | | Plt | Hb | MPV | PDW | M1 | M2 | M2a | M2b | M2c |
| --- | --- | --- | --- | --- | --- | --- | --- | --- | --- | --- |
| Plt | P value | __ | __ | __ | __ | __ | __ | __ | __ | __ |
|  | Rho coefficient | __ | __ | __ | __ | __ | __ | __ | __ | __ |
| Hb | P value | **<0.001** | __ | __ | __ | __ | __ | __ | __ | __ |
|  | Rho coefficient | 0.65 | __ | __ | __ | __ | __ | __ | __ | __ |
| MPV | P value | **<0.001** | **0.003** | __ | __ | __ | __ | __ | __ | __ |
|  | Rho coefficient | -0.76 | -0.5 | __ | __ | __ | __ | __ | __ | __ |
| P  DW | P value | **<0.001** | 0.06 | **<0.001** | __ | __ | __ | __ | __ | __ |
|  | Rho coefficient | -0.56 | -0.33 | 0.65 | __ | __ | __ | __ | __ | __ |
| M1 | P value | 0.053 | **0.03** | 0.3 | **0.004** | __ | __ | __ | __ | __ |
|  | Rho coefficient | -0.35 | -0.39 | 0.2 | 0.49 | __ | __ | __ | __ | __ |
| M2 | P value | **0.03** | **0.02** | 0.07 | **0.004** | **<0.001** | __ | __ | __ | __ |
|  | Rho coefficient | 0.39 | 0.4 | -0.33 | -0.49 | -0.87 | __ | __ | __ | __ |
| M2a | P value | 0.27 | 0.55 | **0.02** | 0.56 | 0.27 | 0.49 | __ | __ | __ |
|  | Rho coefficient | -0.20 | -011 | 0.41 | 0.11 | -0.2 | 0.13 | __ | __ | __ |
| M2b | P value | 0.064 | 0.23 | **0.02** | 0.47 | 0.9 | 0.69 | **<0.001** | __ | __ |
|  | Rho coefficient | 0.33 | 0.22 | -0.42 | -0.13 | -0.004 | 0.07 | -0.88 | __ | __ |
| M2c | P value | 0.42 | 0.15 | 0.08 | 0.42 | 0.24 | **0.03** | **0.02** | 0.07 | __ |
|  | Rho coefficient | 0.15 | 0.26 | -0.31 | -0.15 | -0.22 | 0.39 | -0.41 | 0.33 | __ |

Spearman correlation, P value was significant at <0.05

**Supplementary table (2): Correlation between CD273 and CD274 expressed on the different monocyte-macrophage subtypes and hematological parameters in ITP patients**

| Variable | | Plt | Hb | MPV | PDW | CD273+ M1 | CD274+ M1 | CD273+ M2a | CD274+ M2a | CD273+ M2b | CD274+ M2b | CD273+ M2c | CD274+ M2c |
| --- | --- | --- | --- | --- | --- | --- | --- | --- | --- | --- | --- | --- | --- |
| Plt | P value | __ | __ | __ | __ | __ | __ | __ | __ | __ | __ | __ | __ |
|  | Rho coefficient | __ | __ | __ | __ | __ | __ | __ | __ | __ | __ | __ | __ |
| Hb | P value | **<0.001** | __ | __ | __ | __ | __ | __ | __ | __ | __ | __ | __ |
|  | Rho coefficient | 0.65 | __ | __ | __ | __ | __ | __ | __ | __ | __ | __ | __ |
| MPV | P value | **<0.001** | **0.003** | __ | __ | __ | __ | __ | __ | __ | __ | __ | __ |
|  | Rho coefficient | -0.76 | -0.5 | __ | __ | __ | __ | __ | __ | __ | __ | __ | __ |
| PDW | P value | **<0.001** | 0.06 | **<0.001** | __ | __ | __ | __ | __ | __ | __ | __ | __ |
|  | Rho coefficient | -0.56 | -0.33 | 0.65 | __ | __ | __ | __ | __ | __ | __ | __ | __ |
| CD273+ M1 | P value | 0.53 | **0.05** | 0.6 | 0.23 | __ | __ | __ | __ | __ | __ | __ | __ |
|  | Rho coefficient | -0.11 | -0.35 | 0.1 | -0.22 | __ | __ | __ | __ | __ | __ | __ | __ |
| CD274+ M1 | P value | **0.04** | 0.3 | 0.5 | 0.62 | 0.06 | __ | __ | __ | __ | __ | __ | __ |
|  | Rho coefficient | 0.37 | 0.2 | -0.13 | -0.1 | 0.33 | __ | __ | __ | __ | __ | __ | __ |
| CD273+ M2a | P value | 0.83 | 0.8 | 0.3 | 0.32 | 0.08 | 0.3 | __ | __ | __ | __ | __ | __ |
|  | Rho coefficient | 0.04 | 0.05 | -0.2 | -0.18 | 0.31 | 0.19 | __ | __ | __ | __ | __ | __ |
| CD274+ M2a | P value | 0.7 | 0.93 | 0.9 | 0.22 | 0.17 | 0.8 | **<0.001** | __ | __ | __ | __ | __ |
|  | Rho coefficient | -0.1 | 0.02 | -0.02 | -0.22 | 0.25 | 0.1 | 0.56 | __ | __ | __ | __ | __ |
| CD273+ M2b | P value | **<0.001** | 0.5 | **0.01** | **0.002** | 0.07 | **0.014** | 0.58 | 0.78 | __ | __ | __ | __ |
|  | Rho coefficient | 0.64 | 0.13 | -0.46 | -0.53 | 0.33 | 0.43 | 0.1 | 0.05 | __ | __ | __ | __ |
| CD274+ M2b | P value | **<0.001** | **0.04** | **<0.001** | **0.001** | 0.46 | 0.07 | 0.08 | **0.049** | **<0.001** | __ | __ | __ |
|  | Rho coefficient | 0.7 | 0.36 | -0.6 | -0.55 | 0.13 | 0.32 | 0.32 | 0.35 | 0.64 | __ | __ | __ |
| CD273+ M2c | P value | 0.31 | 0.12 | 0.4 | 0.5 | 0.86 | **0.045** | **0.03** | 0.37 | 0.22 | 0.14 | __ | __ |
|  | Rho coefficient | 0.2 | 0.29 | -0.16 | 0.13 | -0.03 | 0.36 | 0.39 | 0.16 | 0.22 | 0.27 | __ | __ |
| CD274+ M2c | P value | 0.92 | 0.65 | 0.98 | 0.14 | 0.84 | 0.14 | **0.003** | 0.38 | 0.52 | 0.21 | **<0.001** | __ |
|  | Rho coefficient | 0.02 | 0.1 | -0.01 | 0.26 | -0.04 | 0.26 | 0.51 | 0.16 | 0.12 | 0.23 | 0.83 | __ |

Spearman correlation, P value was significant at <0.05
